# Epigallocatechin Gallate Ameliorates the Effects of Prenatal Alcohol Exposure in a Fetal Alcohol Spectrum Disorder-Like Mouse Model

**DOI:** 10.3390/ijms22020715

**Published:** 2021-01-13

**Authors:** Laura Almeida-Toledano, Vicente Andreu-Fernández, Rosa Aras-López, Óscar García-Algar, Leopoldo Martínez, María Dolores Gómez-Roig

**Affiliations:** 1 BCNatal-Barcelona Center for Maternal Fetal and Neonatal Medicine, Hospital Sant Joan de Déu and Hospital Clínic, 08950 Esplugues de Llobregat, Spain; lalmeida@sjdhospitalbarcelona.org (L.A.-T.); ogarciaa@clinic.cat (Ó.G.-A.); 2 Institut de Recerca Sant Joan de Déu, 08950 Esplugues de Llobregat, Spain; 3 Maternal and Child Health and Development Network II (SAMID II), Instituto de Salud Carlos III (ISCIII), 28029 Madrid, Spain; rosam.aras@salud.madrid.org (R.A.-L.); leopoldo.martinez@salud.madrid.org (L.M.); 4 Grup de Recerca Infancia i Entorn (GRIE), Institut d’investigacions Biomèdiques August Pi i Sunyer (IDIBAPS), 08036 Barcelona, Spain; 5 Valencian International University (VIU), 46002 Valencia, Spain; 6 Congenital Malformations Lab, Institute of Medicine and Molecular Genetic (INGEMM), Institute for Health Research of La Paz University Hospital (IdiPAZ), 28046 Madrid, Spain; 7 Department of Neonatology, Hospital Clínic-Maternitat, ICGON, IDIBAPS, BCNatal, 08028 Barcelona, Spain; 8 Department of Pediatric Surgery, Hospital Universitario La Paz, 28046 Madrid, Spain

**Keywords:** FASD-like mouse model, prenatal alcohol exposure (PAE), binge alcohol drinking pattern, moderate alcohol drinking pattern, neural plasticity, angiogenesis, neural maturation, neural differentiation, neurodevelopmental disorders, natural antioxidants, epigallocatechin-3-gallate (EGCG)

## Abstract

Fetal alcohol spectrum disorder is the main preventable cause of intellectual disability in the Western world. Although binge drinking is the most studied prenatal alcohol exposure pattern, other types of exposure, such as the Mediterranean, are common in specific geographic areas. In this study, we analyze the effects of prenatal alcohol exposure in binge and Mediterranean human drinking patterns on placenta and brain development in C57BL/6J mice. We also assess the impact of prenatal treatment with the epigallocatechin-3-gallate antioxidant in both groups. Study experimental groups for Mediterranean or binge patterns: (1) control; (2) ethanol; (3) ethanol + epigallocatechin-3-gallate. Brain and placental tissue were collected on gestational Day 19. The molecular pathways studied were fetal and placental growth, placental angiogenesis (VEGF-A, PLGF, VEGF-R), oxidative stress (Nrf2), and neurodevelopmental processes including maturation (NeuN, DCX), differentiation (GFAP) and neural plasticity (BDNF). Prenatal alcohol exposure resulted in fetal growth restriction and produced imbalances of placental angiogenic factors. Moreover, prenatal alcohol exposure increased oxidative stress and caused significant alterations in neuronal maturation and astrocyte differentiation. Epigallocatechin-3-gallate therapy ameliorated fetal growth restriction, attenuated alcohol-induced changes in placental angiogenic factors, and partially rescued neuronal nuclear antigen (NeuN), (doublecortin) DCX, and (glial fibrillary acidic protein) GFAP levels. Any alcohol consumption (Mediterranean or binge) during pregnancy may generate a fetal alcohol spectrum disorder phenotype and the consequences may be partially attenuated by a prenatal treatment with epigallocatechin-3-gallate.

## 1. Introduction

Alcohol, the most commonly used teratogen, triggers many deleterious effects in the offspring when consumed during pregnancy, with the consequences falling under the umbrella of fetal alcohol spectrum disorder (FASD) [1], of which fetal alcohol syndrome (FAS) is the most severe form of prenatal alcohol exposure (PAE) impairment. FAS is characterized by craniofacial dysmorphology, growth retardation, and central nervous system dysfunctions [1].

Different factors contribute to the pathophysiology of FASD. The teratogenic effects of PAE have great impact on the placenta, the central regulator of the intrauterine environment [2]. FASD in vitro and animal model studies have shown the detrimental effects of PAE on placental size, structure, and function [3] and alterations in the methylation pattern of placental genes affecting its growth and on fetal development [4,5,6]. Moreover, ethanol alters the expression of several placental angiogenic factors, e.g., the vascular endothelial growth factor A (VEGF-A), its receptor VEGFR, and the placental growth factor (PLGF) [7]. Ethanol diffuses through the placenta and rapidly distributes into the fetal compartment, accumulating in the amniotic fluid [8]. The efficiency of the alcohol dehydrogenase isoforms and aldehyde dehydrogenases enzymes expressed by the fetus for alcohol metabolization is limited. Furthermore, CYP2E1 activity, the alternative pathway to metabolize alcohol, is upregulated during development, generating high levels of reactive oxygen species (ROS), which intensify the damage caused by alcohol. In addition, the fetal brain is particularly vulnerable to alcohol due to the downregulation of the antioxidant response in this tissue [9,10]. Thus, PAE affects the central nervous system in all stages of brain development (neurulation, proliferation, migration, differentiation, synaptogenesis, gliogenesis, myelination, apoptosis, and plasticity) through a variety of mechanisms that include oxidative stress and the direct alteration of the epigenetic pattern in the neural lineages [11,12]. Therefore, ethanol may alter the expression of a wide range of neural biomarkers, e.g., the neuronal nuclear antigen (NeuN) [13], doublecortin (DCX) [14], glial fibrillary acidic protein (GFAP) [15], and the brain-derived neurotrophic factor (BDNF) [16], as well as of the oxidative stress biomarkers, e.g., the nuclear factor erythroid 2-related factor 2 (Nrf2) [17].

There is a direct association between the dose and timing of PAE and FAS characteristics [18]. FAS phenotypes are reported in the offspring of mothers who consume high doses of alcohol (acute or binge models) during different stages of pregnancy [19]. Binge drinking is defined by the National Institute on Alcohol Abuse and Alcoholism (NIAAA) as a drinking pattern that brings blood alcohol concentration (BAC) to 0.08 g/dL [20] (this typically occurs with four/five drinks in women/men, respectively, in about two hours) [20]. In Mediterranean countries, mild or moderate patterns of alcohol consumption (chronic model) are more frequently seen, with reduced prevalence of FAS compared to European Eastern countries. However, nearly one out of 14 children diagnosed with FASD were exposed to only one drink per day during their fetal development [21]. Moderate drinking is defined by the NIAAA as up to one/two drinks a day in women/men, respectively [20].

Animal models allow the control of variables linked to FAS phenotypes, e.g., dose, timing, and developmental stage of alcohol exposure during pregnancy. High BACs (≥100 mg/dL), similar to the levels reached in humans [22,23], can be achieved with mouse models of alcohol exposure, to mimic the human binge-drinking pattern. The FAS-like phenotype is easily identified in the mouse offspring when using an alcohol binge-drinking pattern. However, the human moderate drinking pattern is not well established in mouse models, and causal relations between moderate PAE and FASD manifestations are difficult to determine due to the incomplete phenotype and the confounding factors [24].

To date, the evidence indicates there is no safe amount of alcohol consumption during gestation [25]. Furthermore, there is no treatment for FASD, no early intervention for the offspring, and no long-term follow-up to improve behavioral disabilities [26]. FASD research focuses on the underlying mechanisms of ethanol teratogenesis, such as the oxidative stress generated by alcohol metabolism [27]. Moreover, PAE epigenetic changes are the leading cause of the alterations produced by PAE in the neurobiological system [28]. Potential therapies to reduce oxidative stress and epigenetic alterations include natural antioxidants such as epigallocatechin-3-gallate (EGCG), which has been used as a therapeutic tool in oxidative stress-related pathological processes such as cardiovascular diseases, cancer, and Alzheimer’s disease [29]. Recent studies have shown that EGCG administration in fetuses affected by PAE decreases neuronal apoptosis of the rhombencephalon, ameliorates neurogenesis processes, improves fetal growth restriction (FGR), and increases the effect of the endogenous antioxidant defense systems [30]. Additionally, EGCG improves the neuronal plasticity in Down’s syndrome patients, blocking the overexpression of the Dyrk1A protein, a general inhibitor of neuronal plasticity [31].

In this study, we compare the effects of PAE on the placenta and fetal brain development in two humanlike patterns of alcohol exposure, binge and Mediterranean drinking, in a C57BL/6J mouse model. We also describe the effect of EGCG administration on oxidative stress, fetal growth, placental development, and neurogenesis processes in both patterns.

## 2. Results

Two hundred and eighty-three mouse fetuses were included in the study (42 Mediterranean (Med) control, 47 Med ethanol (EtOH), 45 EtOH Med+EGCG, 54 Binge (Bin) control, 44 Bin EtOH, and 47 Bin+EGCG EtOH) from 36 dams. Two hundred and seventy-nine were alive by Day 19 (cesarean section) (one fetal demise in the Med control group, one in the Bin EtOH group, and two in the Bin+EGCG EtOH group). Mean litter size was 8.2 ± 1.9 for the Med control, 7.8 ± 1.3 for the Med EtOH, 7.2 ± 1.9 for the Med+EGCG EtOH, 8.2 ± 1.9 for the Bin control, 7.3 ± 1.6 for the Bin EtOH, and 7.8 ± 1.5 for the EtOH groups. 

### 2.1. Blood Alcohol Concentrations and Epigallocatechin-3-gallate Determination

BACs were first measured from maternal blood samples obtained by cardiac puncture. Table 1 shows BAC determinations under the various experimental conditions. As per the NIAAA definition [20], BACs were above 0.8 g/L in the Bin EtOH and Bin + EGCG EtOH groups. For the Mediterranean groups, BACs were between 0.12 g/L in the Med EtOH and 0.32 in the Med + EGCG EtOH, three to four times less concentrated than for the binge pattern.

EGCG concentrations in plasma were analyzed using ultra-performance liquid chromatography electrospray tandem mass spectrometry (UPLC-ESI-MS/MS) under the various experimental conditions (EGCG alone, Bin EtOH + EGCG, and Med + EGCG EtOH) 40 min after EGCG administration. Similar intergroup results were obtained (mean ± SD: 31.5 µg/mL *±* 7.8 µg/mL) comparable to concentrations reported elsewhere [32].

### 2.2. Fetal Growth

**Figure 1:**
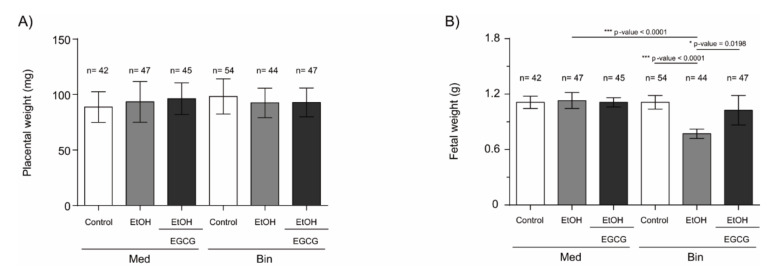
C57BL/6J mouse placental (**A**) and fetal (**B**) weight at gestational Day 19 under the various experimental conditions. Administered once a day: Binge (Bin) control: isocaloric maltodextrin solution (5.52 g/Kg/day); Bin EtOH: 20% (*v/v*) of ethanol solution in tap water (3 g/Kg/day); Bin + EGCG EtOH: ethanol 3 g/Kg/day + EGCG 30 mg/Kg/day. Administered twice a day: Med control, Med: isocaloric maltodextrin solution (1.38 g/Kg/day); Med EtOH: 10% (*v/v*) of ethanol solution (0.75 g/Kg); Med + EGCG EtOH: ethanol 0.75 g/Kg + EGCG 30 mg/Kg. For intergroup comparisons, the Kruskal–Wallis test was used. Asterisks denote the level of significance: * *p*-value < 0.05, *** *p*-value < 0.005

Fetal and placental weight were analyzed at gestational Day 19 to evaluate the impact of binge and Mediterranean drinking patterns on fetal growth, as well as the effects of EGCG coadministration during pregnancy. Figure 1 summarizes fetal and placental weights of the mice under the various experimental conditions. No significant differences in placental weights were found in any of the evaluated groups. However, fetal weights were significantly lower in the Bin EtOH group (*p* < 0.0001) compared to the Bin control or Med EtOH groups (Kruskal–Wallis analyses; *p* < 0.05). Interestingly, similar weights were observed in the Bin + EGCG EtOH and Bin control groups, with significant differences when compared to the Bin EtOH group (*p* < 0.0198), indicating complete recovery of fetal weight in presence of the antioxidant. No fetal weight differences were found for the Mediterranean groups.

### 2.3. Placental Angiogenic Factors

**Figure 2:**
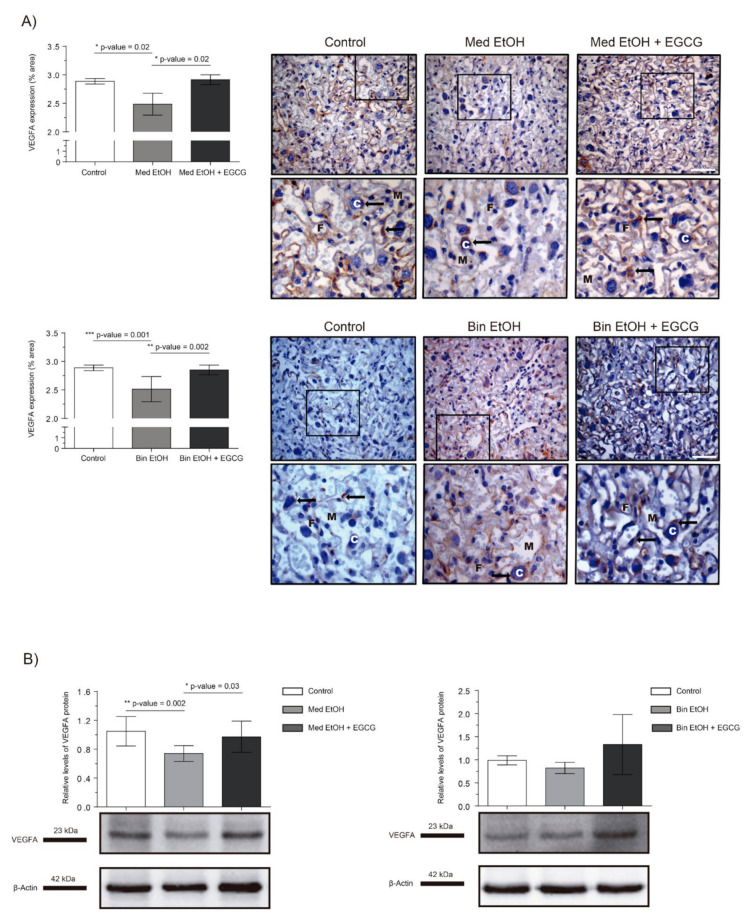
Representative vascular endothelial growth factor A (VEGF-A) immunostaining of placenta sections (**A**) and Western blot VEGF-A protein analysis in placental lysed samples (**B**) for prenatal alcohol exposure (PAE) and EGCG treatment for binge and Mediterranean patterns. (**A**) Boxed VEGF-A immunostaining sections represent the enlarged image of the labyrinth zone. VEGF-A quantification at two different fields (40×) per section. (**B**) Representative Western blot of VEGF-A expression observed in all analyzed samples under the various experimental conditions. Protein levels were normalized using α-tubulin as load control. Arbitrary units express “fold change”. At least five samples from different litters were used from each group. For intergroup comparisons, the Kruskal–Wallis test (Dunn’s test for multiple comparisons) was used. Asterisks denote the level of significance: * *p*-value < 0.05, ** *p*-value < 0.01, *** *p*-value < 0.005. M: maternal blood; F: fetal blood; C: cytotrophoblast; arrows: VEGF-A positive staining.

Immunohistochemistry (IHC) and Western blot (WB) were used to determine the effect of different ethanol doses and EGCG treatment on placental tissue vasculogenesis and angiogenesis. Figure 2 shows the effect of PAE on placental VEGF-A levels. IHC assays (Figure 2A) showed a downregulation of VEGF-A, significant in the Mediterranean (*p* = 0.02) and binge (*p* = 0.001) drinking patterns (Kruskal–Wallis test, Dunn’s correction for multiple comparisons). Additionally, significant increase in VEGF-A levels were observed for the Med EtOH (*p* = 0.02) and Bin EtOH (*p* = 0.002) groups treated with EGCG in comparison to the Med EtOH and the Bin EtOH group, respectively (Figure 2A). WB results were similar to the IHC in both EtOH groups in comparison to the controls; a statistically significant decrease was found for the Med EtOH group (*p* = 0.002) (Figure 2B). As in the IHC, WB measurements showed statistically significant higher expression of VEGF-A in the Med EtOH group treated with EGCG (*p* = 0.03) and a slight trend in the Bin EtOH treated group in comparison to the EtOH groups.

**Figure 3:**
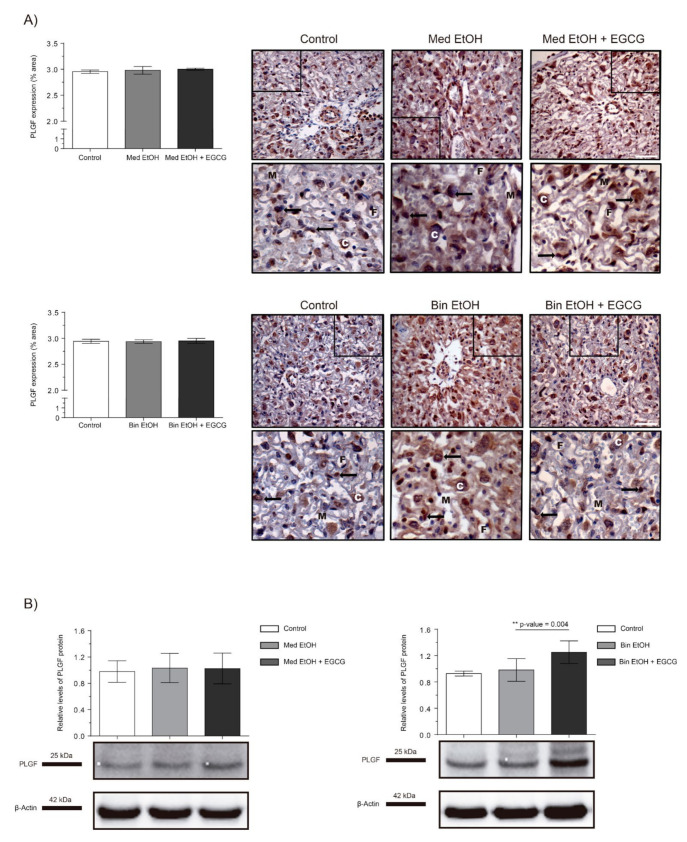
Representative placental growth factor (PLGF) immunostaining in placenta sections (**A**) and Western blot PLGF protein analysis in placental lysed samples (**B**) for PAE and EGCG treatment for binge and Mediterranean patterns. (**A**) Boxed sections in placental growth factor protein immunostaining represent an enlarged image of the labyrinth zone. PLGF protein quantification for two different fields (40×) per section. (**B**) Representative Western blot of PLGF protein expression observed in all samples analyzed under the various experimental conditions. Protein levels were normalized using α-tubulin as load control. Arbitrary units express “fold change”. At least five samples from different litters were used from each group. For intergroup comparisons, the Kruskal–Wallis test (Dunn’s test for multiple comparisons) was used. Asterisks denote the level of significance: ** *p*-value < 0.01. M: maternal blood; F: fetal blood; C: cytotrophoblast; arrows: PLGF positive staining

Figure 3 shows PLGF expression as per IHC and WB analysis of placentas. No significant differences were found in PLGF quantification neither in the immunostaining of placental tissues nor in WB analyses for the experimental groups for the Med EtOH and Bin EtOH groups (Figure 3A,B). However, WB revealed a significant increase in PLGF levels (*p* = 0.004) for the Bin + EGCG EtOH group in comparison to the Bin EtOH group (Figure 3B).

**Figure 4:**
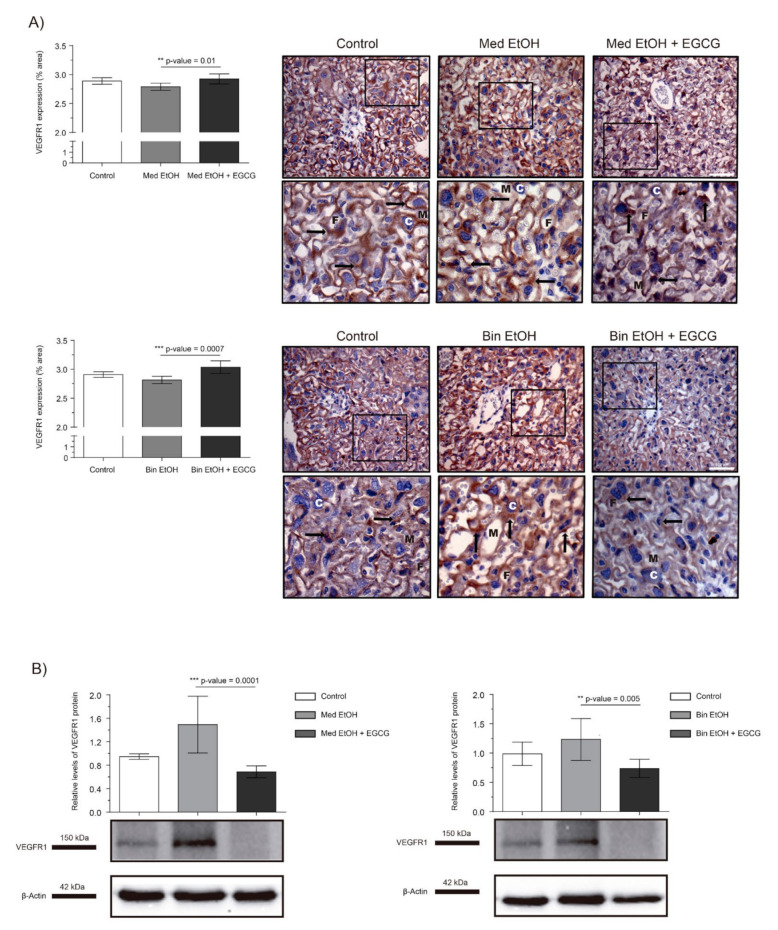
Representative vascular endothelial growth factor receptor 1 (VEGF-R1) immunostaining in placenta sections (**A**) and Western blot VEGF-R1 protein analysis in placental lysed samples (**B**) for PAE and EGCG treatment binge and Mediterranean experimental conditions. (**A**) Boxed sections in VEGF-R1 immunostaining are shown an enlarged image of the labyrinth zone. VEGF-R1 quantification in placental sections in two different fields (40×) per section. (**B**) Representative Western blot of VEGF-R1 expression observed in all samples analyzed for the different experimental conditions. Protein levels were normalized using α-tubulin as load control. Arbitrary units express “fold change”. At least five samples from different litters were used in each group. Kruskal–Wallis test (Dunn’s test for multiple comparisons) was used for inter-group comparisons. Asterisks denote the level of significance: ** *p*-value < 0.01, *** *p*-value < 0.005. M: maternal blood; F: fetal blood; C: cytotrophoblast; arrows VEGF-R1 positive staining.

The effects of PAE on the VEGF-R1 antiangiogenic factor is shown in Figure 4. WB analysis showed an increase in placental VEGF-R1 expression for the Med EtOH and Bin EtOH groups, with no significant differences (Figure 4B). However, EGCG rescued VEGF-R1 expression in the Med EtOH (*p* = 0.0001) and Bin EtOH (*p* = 0.005) groups (Figure 4B). Surprisingly VEGF-R1 expression increased in presence of EGCG in both EtOH groups, as confirmed by the immunostaining results (Figure 4A).

### 2.4. Effect of Ethanol on Oxidative Stress

**Figure 5:**
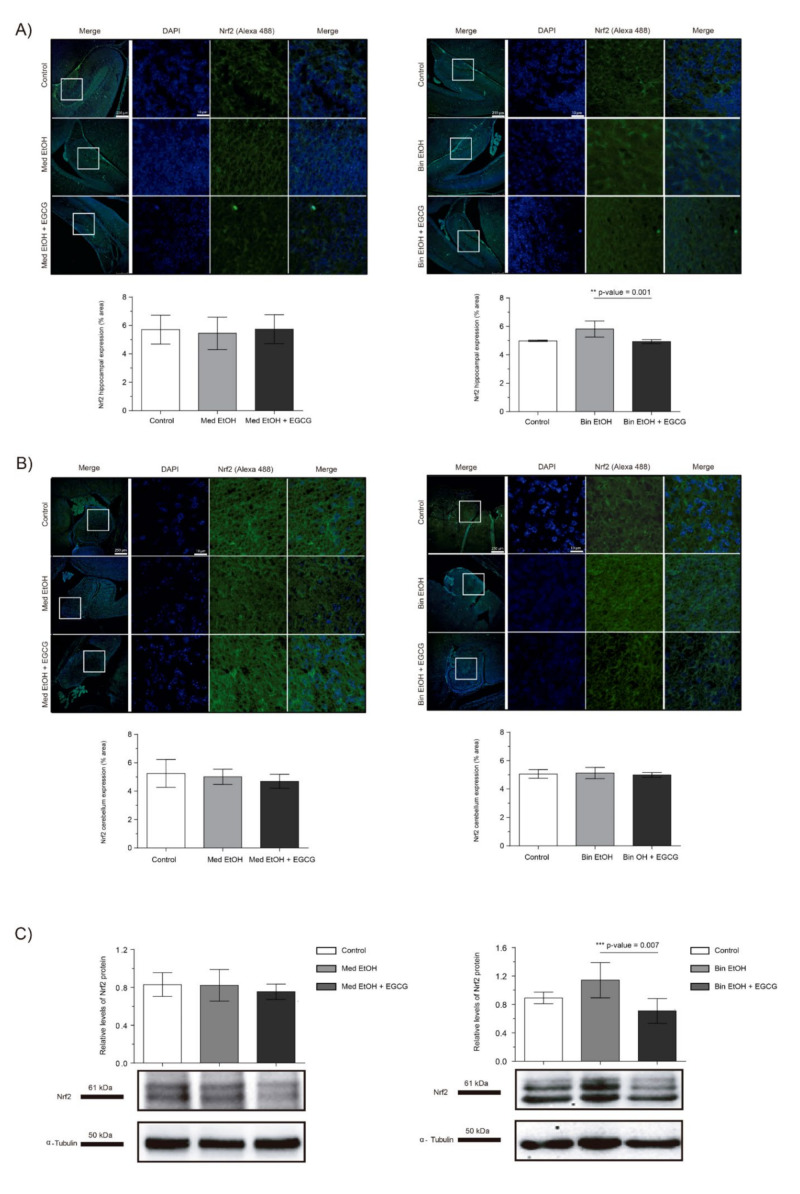
Nuclear factor erythroid 2-related factor 2 (Nrf2) immunofluorescence (Alexa 488 in green for the Nrf2 biomarker, and 4′,6-diamidino-2-phenylindole (DAPI) to stain the nuclei in blue) in the hippocampus (**A**) and cerebellum (**B**). Boxed regions in the hippocampus and cerebellum are shown at higher magnification using 63× Oil Immersion. Nrf2 protein analysis in brain lysed samples (**C**) under the selected experimental conditions (PAE and EGCG treatment). (**A**) Nrf2 quantification in whole sections of the dentate gyrus using a 10× objective lens. (**B**) Nrf2 quantification in whole sections of the cerebellum using a 10× objective lens. (**C**) Representative Western blot of Nrf2 expression analyzed under the various experimental conditions. Protein levels were normalized using α-tubulin as load control. Arbitrary units express “fold change”. At least five samples from different litters were used from each group. For intergroup comparisons, the Kruskal–Wallis test (Dunn’s test for multiple comparisons) was used. Asterisks denote the level of significance: ** *p*-value < 0.01. *** *p*-value < 0.005.

We assessed the effects of PAE and EGCG treatment on fetal brain oxidative stress by measuring the levels of the Nrf2 transcriptional factor, a key regulator of oxidative stress response. Immunofluorescence of the dentate gyrus (DG) of the hippocampus and cerebellum revealed no significant changes in Nrf2 expression in the Med EtOH group; similar results were obtained with WB analysis (Figure 5A–C). However, a slight increase in Nrf2 levels was found in the DG for the Bin EtOH group (Figure 5A) in comparison to the controls, with no statistical significance (Kruskal–Wallis test). Similar results were observed with the WB analysis (Figure 5C): no differences between the Mediterranean groups and a nonsignificant increase in the Bin EtOH group compared to the control. Interestingly, reduced levels of oxidative stress were observed with EGCG treatment in the DG for the Bin EtOH group (*p* = 0.001) (Figure 5A). Similar findings were found with the WB analysis (*p* = 0.007) (Figure 5C).

### 2.5. Neuronal Maturation

**Figure 6:**
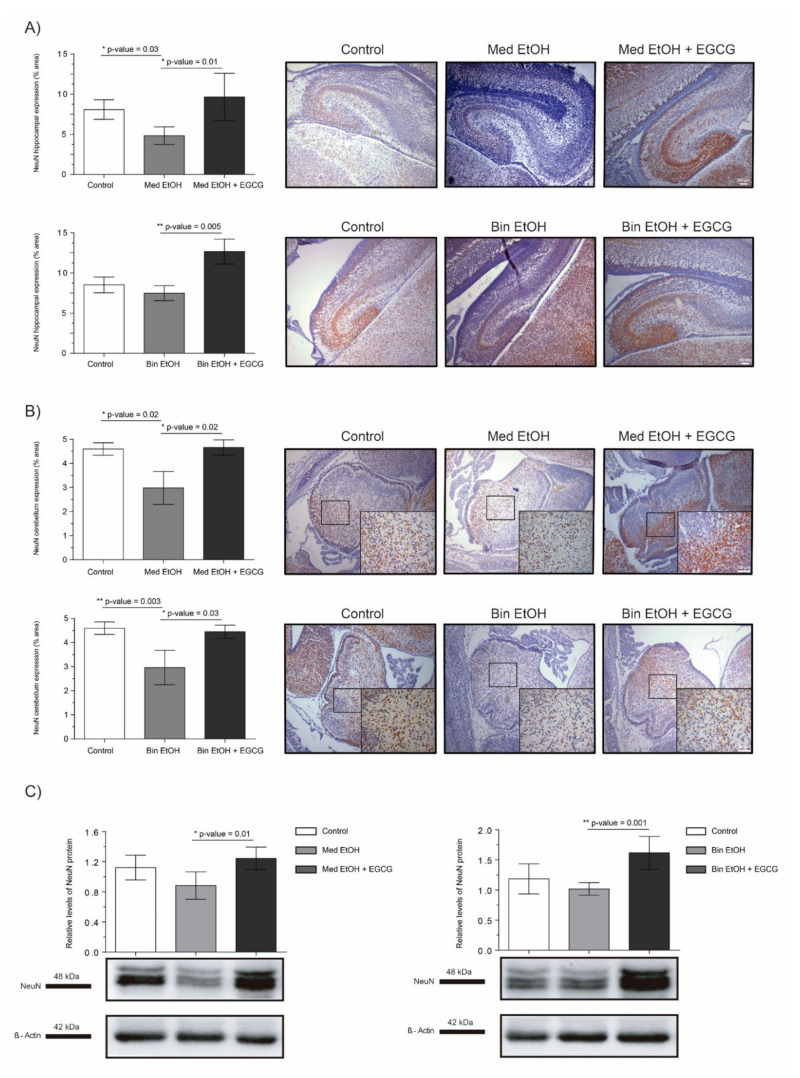
Representative neuronal nuclear antigen (NeuN) immunostaining in the dentate gyrus (**A**) and cerebellum (**B**), and NeuN protein analysis in whole brain lysed samples by Western blot (**C**) for PAE and EGCG treatment for the binge and Mediterranean patterns. (**A**) NeuN^+^ cell quantification in whole sections of the dentate gyrus using a 10× objective lens, (**B**) NeuN^+^ cell quantification in cerebellum sections in two different microscopic fields using a 40× objective lens, and (**C**) Representative Western blot of NeuN expression observed in all analyzed samples under the various experimental conditions. Protein levels were normalized using α-tubulin as load control. Arbitrary units express “fold change”. At least five samples from different litters were used from each group. For intergroup comparisons, the Kruskal–Wallis test (Dunn’s test for multiple comparisons) was used. Asterisks denote the level of significance: * *p*-value < 0.05, ** *p*-value < 0.01.

NeuN and DCX were chosen as representative biomarkers of neural maturation to assess the effects of PAE and EGCG therapy on these processes during fetal brain organogenesis. Similar results in neuronal nuclei quantification were obtained for IHC staining (Figure 6A for DG, Figure 6B for cerebellum) and WB analysis (Figure 6C). PAE caused a decrease of mature neurons in both types of maternal drinking patterns compared to the controls. NeuN^+^ neurons in the DG and in the cerebellum of the Med EtOH group (*p* < 0.05) and in the cerebellum of the Bin EtOH group (*p* < 0.01) were significantly lower than in the control groups (Dunn’s test). Moreover, EGCG treatment in PAE groups led to a recovery of NeuN levels. NeuN expression was significantly higher in the EGCG-treated Med EtOH and Bin EtOH groups compared to the Med EtOH and Bin EtOH groups not treated with EGCG (*p* < 0.05 in all cases).

**Figure 7:**
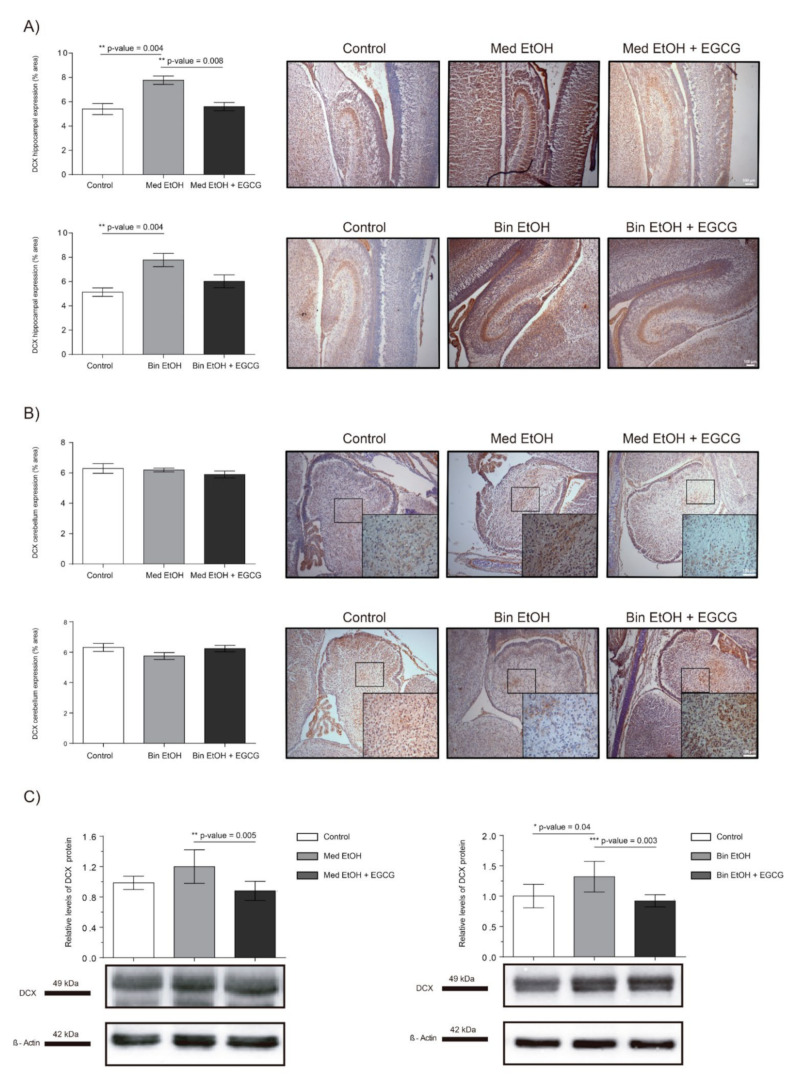
Representative doublecortin (DCX) immunostaining in the hippocampus (**A**) and cerebellum (**B**), and Western blot DCX protein analysis in whole brain lysed samples (**C**) for PAE and EGCG treatment. (**A**) DCX^+^ cell quantification in whole sections of the dentate gyrus using a 10× objective lens. (**B**) DCX^+^ cell quantification in sections of the cerebellum in two different microscopic fields using a 40× objective lens. (**C**) Representative Western blot of DCX expression observed in all analyzed samples for the various experimental conditions. Protein levels were normalized using α-tubulin as load control. Arbitrary units express “fold change”. At least five samples from different litters were used from each group. Kruskal–Wallis test (Dunn’s test for multiple comparisons) was performed for inter-group comparisons. Asterisks denote the level of significance: * *p*-value < 0.05, ** *p*-value < 0.01. *** *p*-value < 0.005.

Representative results of doublecortin (DCX) immunoreactive (IR) cells in the DG and cerebellum are shown in Figure 7, as well as the levels of DCX in whole fetal brain extracts by WB. Immunostaining of the DG of the hippocampus (Figure 7A) showed significant increase of immature neurons in the Med EtOH and Bin EtOH groups compared to the control groups (*p* < 0.005) (Kruskal–Wallis test). DCX levels in the Bin EtOH and Med EtOH groups decreased with EGCG treatment, the latter being statistically significant (*p* <0.05) in comparison to the controls. Quantification of doublecortin-immunoreactive (DCX-IR) neurons in the cerebellum (Figure 7B) revealed no statistical significant differences in any of the experimental groups. According to the WB immunoassay (Figure 7C), quantification of immature neurons was higher for the Bin EtOH and Med EtOH groups compared to the controls, with statistical significance for the Bin EtOH group (*p* < 0.05). Interestingly, the levels of DCX were significantly lower in the EGCG-treated Bin EtOH and Med EtOH groups (*p* < 0.001), indicating a partial recovery of the PAE phenotype. Brains prenatally exposed to alcohol showed an increase of immature neurons, being more pronounced in specific brain areas, such as the DG. This effect may be partially counterbalanced with EGCG treatment.

### 2.6. Astrocyte Differentiation

**Figure 8:**
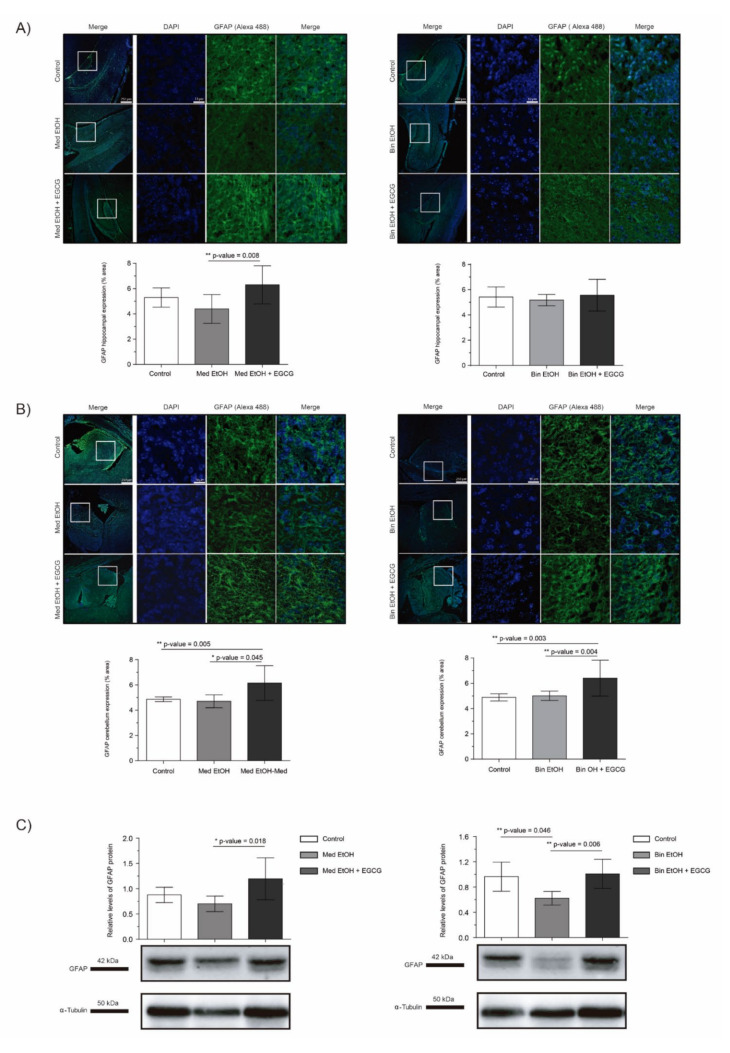
Glial fibrillary acidic protein (GFAP) immunofluorescence (Alexa 488 in green for the GFAP biomarker and DAPI to stain the nuclei in blue) in the hippocampus (**A**) and cerebellum (**B**). Boxed regions in hippocampus and cerebellum are shown at higher magnification using 63× Oil Immersion. Glial fibrillary acidic protein analysis in brain lysed samples (**C**) for PAE and EGCG treatment under the selected experimental conditions. (**A**) GFAP quantification in whole sections of the dentate gyrus using a 10× objective lens. (**B**) GFAP quantification in whole sections of the cerebellum using a 10× objective lens. (**C**) Representative Western blot of GFAP expression analyzed under the various experimental conditions. Protein levels were normalized using α-tubulin as load control. Arbitrary units express “fold change”. At least five samples from different litters were used from each group. For intergroup comparisons, the Kruskal–Wallis test (Dunn’s test for multiple comparisons) was used. Asterisks denote the level of significance: * *p*-value < 0.05, ** *p*-value < 0.01.

The analysis of the glial fibrillary acidic protein (GFAP) allowed to assess glial cell to astrocyte differentiation in PAE fetal brains and the effects of EGCG cotreatment in brains prenatally exposed to alcohol. In spite of the trend towards a reduction of GFAP levels after PAE observed in the DG in WB and immunofluorescence, statistical significance in the results were only confirmed for the Bin EtOH group in comparison to the controls (*p* = 0.046) in WB assays (Figure 8C) (Kruskal–Wallis test). However, a clear and significant increase in the EGCG cotreatment groups (Med EtOH+EGCG and bin EtOH+EGCG) was observed for IHC (DG and cerebellum) (Figure 8A–C), as well as for whole fetal brain WB experiments. Interestingly, GFAP levels were higher in presence of EGCG in comparison to the control groups in these series of experiments (Figure 8A–C).

### 2.7. Neuronal Plasticity

**Figure 9:**
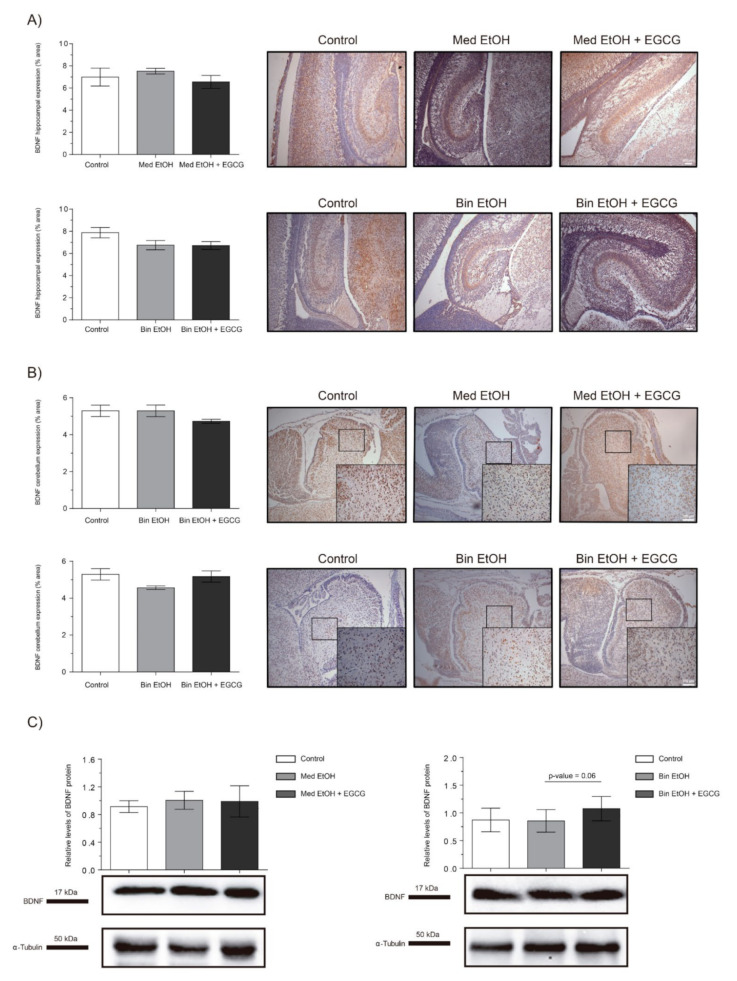
Representative brain-derived neurotrophic factor (BDNF) immunostaining in the hippocampus (**A**) and cerebellum (**B**), and Western blot BDNF protein analysis in whole brain lysed samples (**C**) for PAE and EGCG treatment in the binge and Mediterranean patterns. (**A**) BDNF quantification in the dentate gyrus whole sections using a 10× objective lens. (**B**) BDNF quantification in the cerebellum sections in two different microscopic fields using a 40× objective lens. (**C**) Representative Western blot of BDNF expression observed in all samples analyzed under the various experimental conditions. Protein levels were normalized using α-tubulin as load control. Arbitrary units express “fold change”. At least five samples from different litters were used for each group. For intergroup comparisons, the Kruskal–Wallis test (Dunn’s test for multiple comparisons) was used.

The brain-derived neurotrophic factor (BDNF) is a widely distributed neurotrophin in the central nervous system (CNS) and a classical marker of neural plasticity. We selected the BDNF as biomarker to assess trophic support and synaptic plasticity. Immunostaining of the DG of the hippocampus (Figure 9A) and cerebellum (Figure 9B) showed no statistically significant differences in BDNF levels neither in the EtOH nor the EtOH + EGCG groups in comparison to the controls. Similar results were obtained for the WB analyses (Figure 9C).

## 3. Discussion

Our results support EGCG as a promising antioxidant therapy to attenuate the consequences of prenatal alcohol exposure. Antioxidant therapy may ensure proper development of the placenta and fetal growth. Additionally, the prenatal effect of EGCG on neural maturation and differentiation processes may lead to normal neurodevelopment, improving behavioral and cognitive outcomes in children.

Fetal development is closely linked to placental formation. PAE exerts a negative influence on placentation and leads to FGR [4,33]. In Figure 1, fetuses exposed to high doses of alcohol show lower fetal weights compared to controls, demonstrating the inverse relation between fetal growth and PAE. Our results are in line with previous studies that report FGR on fetuses prenatally exposed to ethanol [33] and the dose-dependent effects of PAE on fetal weight [4]. The expression of angiogenic factors follows dynamic patterns according to the gestational stage to address metabolic requirements. Moreover, PAE produces an imbalance of the expression of these angiogenic factors with the consequent abnormal placental development [34,35]. Downregulation of VEGF-A (Figure 2), a regulator of angiogenesis and vascular permeability, is seen in placentas from fetuses prenatally exposed to ethanol in Mediterranean and binge human drinking patterns. These results reveal the deleterious effect of any type of alcohol consumption during pregnancy on placental angiogenesis. Similarly, decreased levels of placental VEGF with PAE have previously been reported by other authors [34]. Moreover, our study shows an increase in the expression of VEGF-R1, a negative regulator of embryonic angiogenesis, after continued PAE (Figure 4). This increase may be responsible for the abnormal angiogenesis of the placenta. Other authors reported discordances in the expression of VEGF-R1 according to the timing of PAE. Ventureira et al. report similar results to the presented in this study when PAE occurs until GD10 [34], while Lecuyer et al. show a down-regulation of placental VEGF-R1 in ethanol treated mice for the second trimester equivalent [7]. Oxidative stress may be the main cause of the findings [34,36]. Conversely, PLGF is a member of the VEGF family and a key factor in angiogenesis and vasculogenesis during embryogenesis, and is highly regulated depending on the developmental stage [35]. In our study, there are no differences in PLGF levels at GD19 in the different groups after continued PAE (Figure 3). An upregulation of placental PLGF levels in response to PAE in the early stages of pregnancy may promote placental permeability [35] showing a progressive decrease [7] comparable to controls in later stages of pregnancy (when vasculogenesis is completed) such as seen in this work. Alterations in the VEGF-VEGF-R and/or in the VEGF-R1/PLGF ratio produce important imbalances in angiogenesis [37], which are responsible for placental disorders and FGR. In addition, PAE-related oxidative stress also contributes to angiogenesis deregulation and FGR. EGCG (an antioxidant tested in pathologies related to oxidative stress [29]) is a potential therapeutic candidate for FASD. In our study, EGCG ameliorates FGR produced by PAE and attenuates ethanol-related changes in VEGF-A and VEGF-R1 expression, partially restoring the imbalance produced by PAE. Overall, EGCG exerts positive effects on the placenta and fetal growth in all ethanol exposed groups, more evident in the binge drinking group when VEGF-A, VEGF-R, PLGF, and fetal growth were analyzed. In this context, EGCG may be a therapeutic option to maintain adequate vascularization and promote a correct angiogenesis [38] necessary for proper fetal growth.

As previously mentioned, PAE boosts the production of ROS and the dysregulation of the antioxidant systems, being one of the leading causes of FASD physiopathology [17]. Under oxidative stress conditions, Nrf2 is released from its inhibitor Keap-1, translocated from the cytoplasm to the nucleus where it triggers the expression of genes encoding antioxidant proteins and detoxifying enzymes as catalase, superoxide dismutase, and the glutathione peroxidase families [39] by binding to the antioxidant responsive elements located in the promoter region of these detoxifying enzymes. GSK-3β interacts with Nrf2, inhibiting its function and promoting Nrf2 degradation in the oxidative stress delayed response [40] and acting as an Nrf2 regulator when this molecule is accumulated in the cell. In this study, we observe that the intake of high doses of ethanol during fetal development increases the levels of the Nrf2 antioxidant response, being the DG the target for oxidative stress damage. Moreover, EGCG therapy restores Nrf2 expression to levels that are comparable to those of controls. However, no differences are observed in Nrf2 expression in the Med EtOH group (Figure 5). The protective response of Nrf2 towards oxidative stress has been widely studied in adults, but studies in fetal life are limited. A previous study shows increased Nrf2 levels in the brains of mice embryos prenatally exposed to ethanol, similar to those determined in the Bin EtOH group [39]. Moreover, EGCG upregulates Nrf2 expression after different oxidative stress insults [41,42]. However, to date no studies have been performed on the effects of EGCG on Nrf2 in PAE. Our results indicate that with PAE, Nrf2 is upregulated and the presence of EGCG not only does not increase these levels but it also reduces Nrf2 to physiological levels to avoid a pathologic accumulation of Nrf2.

Oxidative stress and the generation of ROS are key factors in FASD neurologic manifestations [43]. In addition to its antioxidant effect, EGCG has neuroprotection properties that include iron-chelation mediated by the hypoxia-inducible factor (HIF-1α) [44] or the induction of neurite outgrowth and differentiation through the protein kinase C pathway [45]. During pregnancy, the CNS has vulnerable periods sensitive to alcohol damage, which affect the developmental processes. Our data reflect the loss of mature neurons (NeuN biomarker, Figure 6), statistically significant in the DG and cerebellum in fetuses at GD19, in both the Mediterranean and binge drinking patterns. Loss of mature neurons in the DG after alcohol exposure has been analyzed in previous studies with contradictory results depending on the moment of ethanol exposure [46,47]. Based on these findings, the timing of exposure during fetal development may be a key factor for neuronal maturation. Our study demonstrates that alcohol-related reduction of mature neurons already occurs during fetal development. Further research is necessary to evaluate neurological and behavioral alcohol-related disorders secondary to the neuronal loss in the DG and cerebellum during fetal development. In the same line, DCX is expressed by neuronal precursor cells and immature neurons in the embryonic and adult brain. Our study shows an increase of DCX+ neurons, particularly in the DG, in the Mediterranean and the binge drinking patterns (Figure 7). Elibol-Can et al. showed similar results in the different hippocampal regions after PAE according to the binge drinking pattern during the second trimester equivalent [46]. In contrast, lower DCX quantification in the DG has been reported in comparison to the controls in adult mice prenatally exposed to ethanol [48]. Increased signaling in immature neurons during fetal life may indicate a delay in the maturation processes produced by the oxidative damage generated by PAE on the organogenesis processes [49,50]. As for the effect of EGCG therapy in fetal neurogenesis, our results show an improvement in maturation and differentiation processes. The expression of NeuN and DCX in the treated groups are comparable to that in the controls (Figure 6 and Figure 7), which indicates that EGCG may exert a beneficial effect on fetal neurogenesis. Studies with natural antioxidants in fetal life are scarce. Similar effects were shown on hippocampal neurogenesis from a neuroinflammation model in adult mice, where EGCG treatment appears to be beneficial [51]. Consistently, a study in ethanol exposed adult mice demonstrated the compensatory effect of EGCG therapy on the affected immature neurons [51]. EGCG also seems to promote proliferation and differentiation, as evidenced by increased Ki67 and neuron specific enolase expression [52,53]. These findings support EGCG as a potential therapeutic compound to prevent the delay in the neurogenesis processes produced by ethanol in fetal life.

Regarding GFAP (a glial cell to astrocyte differentiation biomarker), astrocyte differentiation is reduced following PAE, reaching statistical significance in fetuses exposed to a binge drinking pattern (Figure 8), as reported in previous studies [54]. EGCG therapy elicited a recovery of GFAP to levels comparable to that of controls (Figure 8). Although there are no previous studies on the effect of EGCG on astrocyte differentiation in FASD-like animal models, results from other studies on neurodegenerative diseases show the neuroprotective effect of EGCG on astrocyte differentiation processes [55]. In vitro models also show EGCG neuronal differentiation involvement through the protein kinase C pathway [45], the inhibition of the glycogen synthase kinase-3 (GSK-3) pathway [56], or the modulation of S100B [57].

BDNF is involved in cell survival, development, and function of the CNS, and represents one of the main biomarkers of neuronal plasticity during early development [58]. EGCG has been selected as a potential pharmacological tool against FASD due to its ability to interact and inhibit neuronal plasticity inhibitors such as Dyrk1A [31] and potentiate NGF-induced neurite outgrowth [59,60]. Our study shows no statistically significant differences in BDNF levels after neither PAE nor EGCG administration. In contrast, Feng et al. report a decrease in BDNF levels in rats prenatally exposed to a binge drinking pattern from GD5-GD20, but no differences when alcohol doses are lower [61]. Similarly, Haun et al. show a down-regulation in BDNF expression after the intake of large doses of alcohol in an adult mouse model of four-day ethanol exposure and a significant ethanol consumption decrease in the established model of alcohol dependence after BDNF administration [62]. Our experimental design ensures continued alcohol exposure, which would lead to habituation to ethanol and therefore, no changes in BDNF expression. Further research would be useful to test the effects of EGCG therapy on BDNF expression in FASD-like models with not-continuous ethanol administration.

This article proposes a prenatal intervention in mice exposed to alcohol during development to attenuate the FASD phenotype. One limitation of our study is the experimental design based on continued ethanol exposure during the 19 days of pregnancy. This may lead to an alcohol exposure adaptation in some critical processes related to FASD pathophysiology; however, we consider this continuous 19-day ethanol exposure similar to the alcohol abuse in humans. Additionally, the effects of PAE that contribute to FASD in the third trimester were not considered in this study due to the above-mentioned experimental design.

To the best of our knowledge, this is the first in vivo study that shows the differences in the FASD-like phenotype based on binge and Mediterranean human drinking patterns. Interestingly, any drinking pattern (Mediterranean or binge) produces alcohol related-effects in the offspring. The binge-drinking pattern leads to effects that are more substantial on the different processes, i.e., oxidative stress, neuronal maturation and differentiation, as well as in fetal growth. Nevertheless, the effects of the Mediterranean drinking pattern on angiogenic factors are comparable to binge drinking. Fetal growth is probably preserved in the Med EtOH group because lower doses of ethanol do not reach the threshold to produce a FASD phenotype. Finally, in the present study we evaluate the consequences of PAE based on different patterns of alcohol consumption, as well as the effect of EGCG treatment examining a wide range of FASD manifestations (placental development, fetal growth, angiogenesis, oxidative stress, and neurodevelopmental processes), and show the potential role of EGCG as a pharmacological tool during fetal development.

## 4. Materials and Methods

### 4.1. Animals, Housing, and Ethical Statement

Eight-week-old male (*n* = 20) and female (*n* = 45) C57BL/6J mice were purchased from Charles River (Barcelona, Spain) and housed in the facilities of Sant Joan de Déu Hospital, University of Barcelona. Animals were housed in standard conditions at 21 ± 1 °C, 55±10 % relative humidity under a controlled 12 h light/dark cycle and had free access to water and standard chow. Animal procedures were approved by the Animal Experimental Ethics Committee of the University of Barcelona (23 January 2016) and were registered on the Generalitat de Catalunya, Serveis Territorials d’Agricultura, Ramaderia, Pesca i Alimentació a Barcelona (identification code: 713/15-8744) and carried out in accordance with the recommendations in the ARRIVE guidelines for the care and use of experimental animals and the U.K. Animals (Scientific Procedures) Act, 1986 and EU Directive 2010/63/EU for animal experiments.

### 4.2. Prenatal Alcohol Exposure and Epigallocatechin-3-Gallate Treatment

Pure absolute ethanol (ethyl alcohol, EtOH, 1000 mL) was provided from PanReac AppliChem ITW Reagents (Dublin, Ireland), maltodextrin (100% Maltodextrin Powder, Pure series^®^) was purchased from Bulk Powders (Essex, UK) and EGCG (Teavigo^®^, 94% EGCG, 150 mg, 60 Count) was provided by Healthy Origins (Pittsburgh, PA, USA).

**Figure 10:**
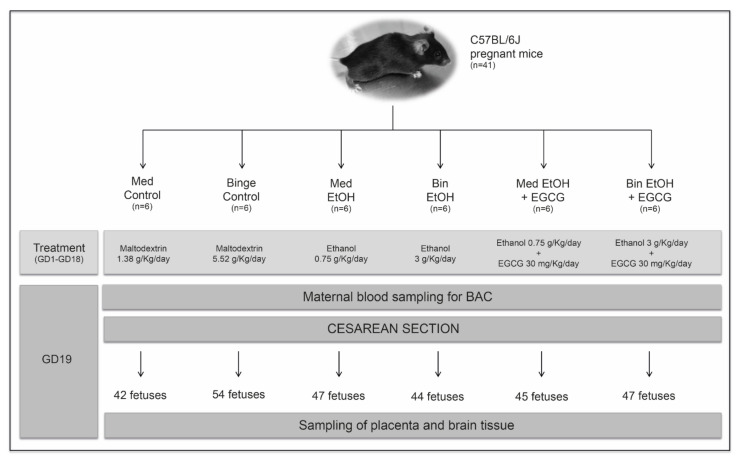
Flowchart of the experimental design. Sample size and doses of maltodextrin, alcohol, and epigallocatechin-3-gallate are specified for each experimental group. Maternal blood sampling to determine blood alcohol concentration and birth by cesarean section were obtained on gestational Day 19. The gestational period of the mice corresponds to the first and second human equivalent. EtOH: ethanol; Bin: binge; Med: Mediterranean; EGCG: epigallocatechin-3-gallate; GD: gestational day; BAC: blood alcohol concentration.

Mice were left to acclimatize, and then matched 2:1 (female:male). Pregnant mice (determined by observing sperm plugs) were individually housed in standard plastic cages to avoid any additional stress, and randomly allocated to one of the six experimental groups: (1) Mediterranean (Med) control: isocaloric maltodextrin solution (1.38 g/Kg/day) given in two administrations (eight-hour dosing interval); (2) EtOH Med pattern: 10% (*v*/*v*) of an ethanol solution (1.5 g/Kg/day) in two administrations (eight-hour dosing interval); (3) EtOH Med and EGCG: ethanol (1.5 g/Kg/day) in two administrations (eight-hour dosing interval) and EGCG (30 mg/Kg/day) once a day with the first dose of ethanol; (4) Binge (Bin) control: isocaloric maltodextrin solution (5.52 g/Kg/day) once a day; (5) EtOH Bin pattern: 20% (*v*/*v*) of ethanol solution in tap water (3 g/Kg/day) once a day; (6) EtOH Bin and EGCG (ethanol 3 g/Kg/day and EGCG 30 mg/Kg/day) once a day. All administrations were performed via oral gavage. An isocaloric maltodextrin solution equivalent to the calorie intake of alcohol was administered to each control group. All animals had free access to water. At Day 19 of pregnancy, mice were terminally anesthetized with pentobarbital, maternal blood samples obtained using cardiac puncture, and fetuses delivered by cesarean section. Fetal and placental weights were obtained before collecting placental and brain tissue. All tissue samples were frozen in liquid nitrogen prior their storage at −80 ℃. The design of the study is summarized in Figure 10.

### 4.3. Reagents and Antibodies

VEGF-A (ref. SC-7269, 21 kDa monomer) was purchased from Quimigen SL (Madrid, Spain); PLGF (ref. ab180734, 25 kDa), VEGFR (ref. ab32152, 150 kDa (Flt1), NeuN (ref. ab177487, 48.5 kDa), doublecortin (ref. ab135349, 49 kDa), GFAP (ref. ab7260, 55 kDa), and BDNF (ref. AB226843, 17 kDa) from Abcam (Cambridge, MA, USA); Nrf2 (ref. sc-722, 61 and 68 kDa) from Santa Cruz Biotechnology, Inc. (Dallas, TX, USA); Alfa-tubulin (ref. T8203, dil 1:2000, 50 kDa), beta-actin (ref. A3854, 42 kDa, dil 1:2500), and Anti Rabbit IgG secondary (ref. A0545, dil 1:2000) from Sigma-Aldrich (Sant Louis, Missouri, USA), and goat antimouse IgG (ref. G21040, dil 1:10,000) from Thermo Fisher Technologies (Waltham, MA, USA).

### 4.4. Blood Alcohol Concentration and Epigallocatechin-3-Gallate Determination in Pregnant Mice

One milliliter of maternal blood was collected in heparin BD Vacutainers^®^ by intracardiac puncture 45 min post-treatment. Samples were incubated at room temperature for five minutes and then centrifuged at 1750× *g* at 4 °C for 20 min. Serum was isolated and BAC measured using the Ethanol Assay Kit (MAK076) purchased from Sigma-Aldrich. A master reaction mix (50 µL per well) containing two microliters of serum sample was performed following the recommendations of the supplier. Wells were mixed using a horizontal shaker and the reactions were incubated for 30 min at 37 °C or 60 min in the dark. Finally, measurements of absorbance at 570 nm were carried out.

The remaining serum was stored at −80 °C to evaluate EGCG concentration. For analytical purposes aliquots of serum containing EGCG (0.350 mL) were stored in Eppendorf LoBind tubes (Sarstedt 72,706,600) at −80 °C containing 20 μL of vc-EDTA (1.38 g NaH_2_PO_4_: H_2_O, 5 g ascorbic acid, and 25 mg EDTA in 25 mL H_2_O miliQ, pH 3.8) until further analysis. Samples were analyzed by the department of pharmacology of Institut Hospital del Mar d’Investigacions Mèdiques (Barcelona, Spain) following a previously published methodology [63].

### 4.5. Western Blot Analysis

Protein extractions were performed by mechanical tissue rupture using the Politron device (Omni Tissue Homogenizer, Omni International, Kennesaw, GA, USA) after introducing the samples in RIPA buffer (Life Technologies S.A, 89900, Carlsbad, CA, USA), using three cycles of 30 s per sample. Samples were then quantified with the DC Protein Assay kit (Bio-rad Laboratories S.A., Madrid, Spain) and absorbance measured at 780 nm (the Lowry test). After quantification, 40 µg of total protein were loaded in a volume of 30 µL per well in RIPA buffer by adding 6 μL of Loading Buffer 5× (3.125 mL 1 M Tris-HCl (pH = 6.8), 5.75 mL glycerol 87%, 1 g SDS, 1 mL β-mercaptoethanol, and 1 mL 5% bromophenol blue), heating the samples on a thermoblock (Thermo Scientific, Waltham, MA, USA) at 95 °C for protein denaturation. Electrophoresis was then performed on a 12% acrylamide gel using the molecular weight marker (precision plus protein dual color standard from BioRad, 1610374). An electric current was next applied to the electrophoresis cuvette with running buffer (3.03 g/L of Tris Base, 1.44 g/L of glycine, 1 g/L of SDS). Next, proteins were transferred to a polyvinylidene fluoride membrane (Bio-Rad Laboratories SA, 162-0177) that had been previously activated five minutes in methanol, five minutes in distilled H_2_O, and five minutes in transfer buffer (3.03 g/L of Tris-Base, 14.4 g/L of glycine and 200 mL/L of methanol). Protein transfer was performed in transfer buffer at 4 °C in a cold chamber for two to three hours depending on the molecular weight of the proteins. Subsequently, three five-minute washes with tris-buffered saline (TBS-T) were performed (2.4 g/L Tris-HCl pH = 7.6 and 8.8 g/L NaCl and 1 mL Tween 20); membranes were blocked with BSA 5% diluted in TBS-T for 30 min. Finally, the membranes were incubated with the primary antibody overnight at 4 °C with stirring; 1:1000 dilutions in BSA 2% were used for primary antibody incubation. The next day, the primary antibody was collected and membranes washed three times (for five minutes) with TBS-T; the secondary antimouse or antirabbit antibody was then added for two hours at room temperature with stirring. Membranes were developed with a 1: 1 mixture of the Pierce ECL WB Substrate (Cultek S.L., Madrid, Spain) in the dark using the iBright CL1000 (Invitrogen, Thermo Fisher Scientific, Cornellà de Llobregat, Spain) device. Densitometric analysis was performed to determine the intensity of the bands using the Image J program. The intensity values obtained from the quantification were normalized with respect to the values obtained from the bands of the control protein (tubulin or actin), expressed at equal levels in all situations.

### 4.6. Immunohistochemistry

Fetal brain and placenta samples were fixed in 10% buffered formalin and next embedded in paraffin. Sagittal 5-μm-thick brain and transversal placenta sections were prepared, mounted on glass slides, and allowed to dry. They were next deparaffinized, unmasked, and peroxidase blocked before applying the primary antibodies. The following primary antibodies were used at the indicated dilutions: anti-VEGF-A (1:1000), anti-PLGF (1:100), anti-VEGF-R (1:200), anti-NeuN (1:700), anti-DCX (1:1500), and anti-BDNF (1:250). Slides were incubated overnight at 4 °C and then washed and incubated with the secondary horseradish peroxidase (HRP) conjugated antibodies: anti-IgG-rabbit (1:200) and anti-IgG-mouse (1:200) for one hour at room temperature. Finally, slides were visualized with diaminobenzidine and lightly counterstained with hematoxylin before being dehydrated, cleared, and mounted. The same steps were followed without the primary antibodies for negative controls; no staining was observed. Staining specificity was established by staining run simultaneously for each slide under the six experimental conditions. VEGF-A, PLGF and VEGF-R counts were carried out by selecting two center areas in the labyrinth; NeuN, and DCX counts were carried out by selecting two center areas in the cerebellar vermis and a whole area in the DG of the hippocampus from 6–10 different samples for each group. Images were captured with an Olympus light microscope using 10× and 40× magnifications. Images were obtained with a specific image software (Image ProPlus) and quantification carried out using an Image J Analysis Software and a color deconvolution algorithm to determine the percentage of positive immunostaining. Immunohistochemical staining was analyzed establishing a positive area/total area ratio (%).

### 4.7. Immunofluorescence

Five-micrometer-thick brain sections were prepared, mounted on glass slides, and allowed to dry. Slides were next deparaffinized, unmasked, and peroxidase blocked before applying the primary antibodies to Nrf2 (1:250) or GFAP (1:250) for one hour at room temperature. The following steps were performed in the dark: slides were incubated with the secondary antibody, a goat antirabbit IgG, coupled to Alexa Fluor-488 (1:1000) for one hour at room temperature; next, they were mounted and the nucleus stained using VectashieldR Antifade Mounting Medium with DAPI. Immunofluorescence was performed simultaneously for all histological sections of each antibody and pictures acquired the same day. Image acquisition conditions were set according to the brightest sample (exposure time, contrast and color balance) for all pictures. Nrf2 and GFAP counts were carried out by selecting two center areas in the cerebellar vermis and a whole area in the DG of the hippocampus from 6–10 different samples in each group. Images were captured using the same imaging settings for all experimental conditions and acquired with the confocal microscope Leica TCS SP5 (Leica Microsistemas S.L.U., objective 10 and 63x). GFAP and Nrf2 signals were quantified using the Image J Analysis Software. The following quantification settings for all immunofluorescence images were used: background correction was performed selecting a region of interest (ROI) in the background applying the command Proces/Math/Subtract, which subtracted the mean of the ROI plus an additional value equal to the standard deviation of the ROI multiplied by 3; the intensity of fluorescence quantification was then measured using the command Analyze/Measure and limiting to a threshold of 100 to 255 for Nrf2 and GFAP quantifications. For immunofluorescence evaluation, cerebellum and DG were analyzed establishing a positive fluorescence signal area/total area ratio (% area).

### 4.8. Statistical Analyses

Database management and statistical analyses of the variables were performed using the SPSS v.22 (IBM, Armonk, NY, USA) and the GraphPad (Prism, San Diego, CA, USA) software v.6.0. For descriptive statistics, means and standard deviations (SD) were used. Intergroup comparisons were performed using the non-parametrical Kruskal–Wallis test (Dunn’s correction for multiple comparisons) to analyze the differences in placental and brain protein expression. Statistical significance was set at *p* < 0.05 for all the analyses. At least three different experiments were performed to obtain the mean for each sample and at least five different samples from different litters were used in the statistical analyses.

## 5. Conclusions

The harmful effects of ethanol on fetal development are well known. However, few studies compare the consequences of ethanol intake considering the different patterns of human alcohol consumption. In this study, we analyze the effects of PAE on fetal growth, placenta, and neurodevelopment and show that no amount of alcohol may be considered safe during pregnancy. The timing of ethanol exposure is a key factor in FASD pathophysiology. PAE promotes alterations in placental angiogenesis, fetal growth restriction, and disorders in neuronal maturation and astrocyte differentiation processes. Furthermore, the binge drinking pattern generates the most harmful effects on the fetus. Finally, EGCG as a potential antioxidant, appears to be a safe nutraceutical option to ameliorate FASD manifestations in exposed individuals. EGCG reduces the oxidative stress generated by alcohol exposure mitigating its teratogenic effects. More studies are needed to evaluate the possible beneficial effects of the antioxidant therapy on fetal angiogenesis and neurogenesis, and the molecular pathways related to these outcomes. Finally, the results of this study need to be tested in humans to translate these findings to women that drink alcohol during pregnancy.

## Figures and Tables

**Table 1:** Blood alcohol concentrations for the various experimental conditions.

Condition	Sample Size	nmol/uL (Mean ± SD)	g/L (Mean ± SD)
**Med EtOH**	4	2.69 ± 0.80	0.12 ± 0.04
**Med + EGCG EtOH**	5	6.94 ± 2.36	0.32 ± 0.11
**Bin EtOH**	4	24.03 ± 1.64	1.11 ± 0.08
**Bin + EGCG EtOH**	4	32.22 ± 7.24	1.48 ± 0.33

EtOH Med: 10% (*v*/*v*) of ethanol solution in tap water (0.75 g/Kg) in two administrations; Med + EGCG EtOH: ethanol 0.75 g/Kg + EGCG 30 mg/Kg in two administrations; Bin EtOH: 20% (*v*/*v*) of ethanol solution (3 g/Kg/day) once a day; Bin + EGCG EtOH: ethanol 3 g/Kg/day (30 mg/Kg/day) once a day. Med: Mediterranean pattern; Bin: binge pattern; EtOH: ethanol; EGCG: epigallocatechin-3-gallate; SD: standard deviation.

## Data Availability

The data presented in this study are available on request from the corresponding author.

## References

[1] Hoyme H.E., Kalberg W.O., Elliott A.J., Blankenship J., Buckley D., Marais A.-S., Manning A.M., Robinson K.L., Adam P.M., Abdul-Rahman O. (2016). Updated Clinical Guidelines for Diagnosing Fetal Alcohol Spectrum Disorders. Pediatrics.

[2] Carter R.C., Wainwright H., Molteno C.D., Georgieff M.K., Dodge N.C., Warton F., Meintjes E.M., Jacobson J.L., Jacobson S. (2016). Alcohol, Methamphetamine, and Marijuana Exposure Have Distinct Effects on the Human Placenta. Alcohol Clin. Exp. Res..

[3] Burd L., Roberts D., Olson M., Odendaal H. (2007). Ethanol and the placenta: A review. J. Matern. Neonatal. Med..

[4] Gundogan F., Gilligan J., Qi W., Chen E., Naram R., De La Monte S.M. (2015). Dose effect of gestational ethanol exposure on placentation and fetal growth. Placenta.

[5] Rosenberg M.J., Wolff C.R., El-Emawy A., Staples M.C., Perrone-Bizzozero N.I., Savage D.D. (2010). Effects of moderate drinking during pregnancy on placental gene expression. Alcohol.

[6] Joya X., Salat-Batlle J., Velezmoro-Jáuregui G., Clavé S., Garcia-Algar O., Vall O. (2015). Prenatal ethanol exposure and placental hCG and IGF2 expression. Placenta.

[7] Lecuyer M., Laquerrière A., Bekri S., Lesueur C., Ramdani Y., Jégou S., Uguen A., Marcorelles P., Marret S., Gonzalez B.J. (2017). PLGF, a placental marker of fetal brain defects after in utero alcohol exposure. Acta Neuropathol. Commun..

[8] Clarke D.W., Steenaart N.A., Breedon T.H., Brien J.F. (1985). Differential pharmacokinetics for oral and intraperitoneal administration of ethanol to the pregnant guinea pig. Can. J. Physiol. Pharmacol..

[9] Agarwal D.P. (2001). Genetic polymorphisms of alcohol metabolizing enzymes. Pathol. Biol..

[10] Sanchis R., Guerri C. (1986). Alcohol-metabolizing enzymes in placenta and fetal liver: Effect of chronic ethanol intake. Alcohol Clin. Exp. Res..

[11] Goodlett C.R., Horn K.H., Zhou F.C. (2005). Alcohol teratogenesis: Mechanisms of damage and strategies for intervention. Exp. Biol. Med..

[12] Almeida L., Andreu-Fernández V., Navarro-Tapia E., Aras-López R., Serra-Delgado M., Martínez L., Garcia-Algar O., Gomez-Roig M.D.M. (2020). Models for the Study of Fetal Alcohol Spectrum Disorders: An Overview. Front. Pediatrics.

[13] Powrozek T.A., Zhou F.C. (2005). Effects of prenatal alcohol exposure on the development of the vibrissal somatosensory cortical barrel network. Dev. Brain Res..

[14] Sari Y., Zhang M., Mechref Y. (2010). Differential expression of proteins in fetal brains of alcohol-treated prenatally C57BL/6 mice: A proteomic investigation. Electrophoresis.

[15] Amiri S., Davie J.R., Rastegar M. (2019). Chronic Ethanol Exposure Alters DNA Methylation in Neural Stem Cells: Role of Mouse Strain and Sex. Mol. Neurobiol..

[16] Ceccanti M., Mancinelli R., Tirassa P., Laviola G., Rossi S., Romeo M., Fiore M. (2012). Early exposure to ethanol or red wine and long-lasting effects in aged mice. A study on nerve growth factor, brain-derived neurotrophic factor, hepatocyte growth factor, and vascular endothelial growth factor. Neurobiol. Aging.

[17] Shanmugam S., Patel D., Wolpert J., Keshvani C., Liu X., Bergeson S.E., Kidambi S., Mahimainathan L., Henderson G.I., Narasimhan M. (2019). Ethanol Impairs NRF2/Antioxidant and Growth Signaling in the Intact Placenta In Vivo and in Human Trophoblasts. Biomolecules.

[18] Astley S.J. (2013). Validation of the fetal alcohol spectrum disorder (FASD) 4-Digit Diagnostic Code. J. Popul. Ther. Clin. Pharmacol..

[19] O’Leary C.M., Bower C. (2012). Guidelines for pregnancy: What’s an acceptable risk, and how is the evidence (finally) shaping up?. Drug Alcohol Rev..

[20] National Institute on Alcohol Abuse and Alcoholism (NIAAA) (2017). Drinking Levels Defined.

[21] Kesmodel U.S., Nygaard S.S., Mortensen E.L., Bertrand J., Denny C.H., Glidewell A., Astley Hemingway S. (2019). Are Low-to-Moderate Average Alcohol Consumption and Isolated Episodes of Binge Drinking in Early Pregnancy Associated with Facial Features Related to Fetal Alcohol Syndrome in 5-Year-Old Children?. Alcohol Clin. Exp. Res..

[22] Thiele T.E., Crabbe J.C., Boehm S.L. (2014). “Drinking in the dark” (DID): A simple mouse model of binge-like alcohol intake. Curr. Protoc. Neurosci..

[23] Carson E.J., Pruett S.B. (1996). Development and characterization of a binge drinking model in mice for evaluation of the immunological effects of ethanol. Alcohol Clin. Exp. Res..

[24] Becker H.C., Diaz-Granados J.L., Randall C.L. (1996). Teratogenic actions of ethanol in the mouse: A minireview. Pharmacol. Biochem. Behav..

[25] Charness M.E., Riley E.P., Sowell E.R. (2016). Drinking During Pregnancy and the Developing Brain: Is Any Amount Safe?. Trends Cogn. Sci..

[26] Wilhoit L.F., Scott D.A., Simecka B.A. (2017). Fetal Alcohol Spectrum Disorders: Characteristics, Complications, and Treatment. Community Ment. Health J..

[27] Joya X., Garcia-Algar O., Salat-Batlle J., Pujades C., Vall O. (2015). Advances in the development of novel antioxidant therapies as an approach for fetal alcohol syndrome prevention. Birth Defects. Res. Part A Clin. Mol. Teratol..

[28] Lussier A.A., Bodnar T.S., Mingay M., Morin A.M., Hirst M., Kobor M.S., Weinberg J. (2018). Prenatal Alcohol Exposure: Profiling Developmental DNA Methylation Patterns in Central and Peripheral Tissues. Front Genet..

[29] Zaveri N.T. (2006). Green tea and its polyphenolic catechins: Medicinal uses in cancer and noncancer applications. Life Sci..

[30] Zhang Y., Wang H., Li Y., Peng Y. (2018). A review of interventions against fetal alcohol spectrum disorder targeting oxidative stress. Int. J. Dev. Neurosci..

[31] De la Torre R., de Sola S., Hernandez G., Farré M., Pujol J., Rodriguez J., Espadaler J.M., Langohr K., Cuenca-Royo A., Principe A. (2016). Safety and efficacy of cognitive training plus epigallocatechin-3-gallate in young adults with Down’s syndrome (TESDAD): A double-blind, randomised, placebo-controlled, phase 2 trial. Lancet Neurol..

[32] Li J., Zeng J., Peng J., Jia Y., Li C.M. (2019). Simultaneous determination of the pharmacokinetics of A-type EGCG and ECG dimers in mice plasma and its metabolites by UPLC-QTOF-MS. Int. J. Food Sci. Nutr..

[33] Lo J.O., Schabel M.C., Roberts V.H.J., Wang X., Lewandowski K.S., Grant K.A., Frias A.E., Kroenke C.D. (2017). First trimester alcohol exposure alters placental perfusion and fetal oxygen availability affecting fetal growth and development in a non-human primate model. Am. J. Obstet. Gynecol..

[34] Ventureira M.R., Sobarzo C., Argandoña F., Palomino W.A., Barbeito C., Cebral E. (2019). Decidual vascularization during organogenesis after perigestational alcohol ingestion. Reproduction.

[35] Haghighi Poodeh S., Salonurmi T., Nagy I., Koivunen P., Vuoristo J., Räsänen J., Sormunen R., Vainio S., Savolainen M. (2012). Alcohol-induced premature permeability in mouse placenta-yolk sac barriers in vivo. Placenta.

[36] Coll T.A., Chaufan G., Pérez-Tito L.G., Ventureira M.R., de Molina M.D.C.R., Cebral E. (2018). Cellular and molecular oxidative stress-related effects in uterine myometrial and trophoblast-decidual tissues after perigestational alcohol intake up to early mouse organogenesis. Mol. Cell. Biochem..

[37] Helske S., Vuorela P., Carpén O., Hornig C., Weich H.H.E. (2001). Expression of vascular endothelial growth factor receptors 1, 2 and 3 in placentas from normal and complicated pregnancies. Mol. Hum. Reprod..

[38] Morbidelli L., Terzuoli E., Donnini S. (2018). Use of nutraceuticals in angiogenesis-dependent disorders. Molecules.

[39] Dong J., Sulik K.K., Chen S.Y. (2008). Nrf2-mediated transcriptional induction of antioxidant response in mouse embryos exposed to ethanol in vivo: Implications for the prevention of fetal alcohol spectrum disorders. Antioxid. Redox Signal..

[40] Jain A.K., Jaiswal A.K. (2007). GSK-3β acts upstream of Fyn kinase in regulation of nuclear export and degradation of NF-E2 related factor 2. J. Biol. Chem..

[41] Han X.D., Zhang Y.Y., Wang K.L., Huang Y.P., Yang Z.B., Liu Z. (2017). The involvement of Nrf2 in the protective effects of (-)-Epigallocatechin-3-gallate (EGCG) on NaAsO2-induced hepatotoxicity. Oncotarget.

[42] Sun W., Liu X., Zhang H., Song Y., Li T., Liu X., Liu Y., Guo L., Wang F., Yang T. (2017). Epigallocatechin gallate upregulates NRF2 to prevent diabetic nephropathy via disabling KEAP1. Free Radic. Biol. Med..

[43] Wells P.G., Bhatia S., Drake D.M., Miller-Pinsler L. (2016). Fetal oxidative stress mechanisms of neurodevelopmental deficits and exacerbation by ethanol and methamphetamine. Birth Defects Res. Part C Embryo Today Rev..

[44] Siddiq A., Ayoub I.A., Chavez J.C., Aminova L., Shah S., LaManna J.C., Patton S.M., Connor J.R., Cherny R.A., Volitakis I. (2005). Hypoxia-inducible factor prolyl 4-hydroxylase inhibition: A target for neuroprotection in the central nervous system. J. Biol. Chem..

[45] Reznichenko L., Amit T., Youdim M.B.H., Mandel S. (2005). Green tea polyphenol (-)-epigallocatechin-3-gallate induces neurorescue of long-term serum-deprived PC12 cells and promotes neurite outgrowth. J. Neurochem..

[46] Elibol-Can B., Dursun I., Telkes I., Kilic E., Canan S., Jakubowska-Dogru E. (2014). Examination of age-dependent effects of fetal ethanol exposure on behavior, hippocampal cell counts, and doublecortin immunoreactivity in rats. Dev. Neurobiol..

[47] Klintsova A.Y., Helfer J.L., Calizo L.H., Dong W.K., Goodlett C.R., Greenough W.T. (2007). Persistent Impairment of Hippocampal Neurogenesis in Young Adult Rats Following Early Postnatal Alcohol Exposure. Alcohol Clin. Exp. Res..

[48] Olateju O.I., Spocter M.A., Patzke N., Ihunwo A.O., Manger P.R. (2018). Hippocampal neurogenesis in the C57BL/6J mice at early adulthood following prenatal alcohol exposure. Metab. Brain Dis..

[49] Shnitko T.A., Liu Z., Wang X., Grant K.A., Kroenke C.D. (2019). Chronic Alcohol Drinking Slows Brain Development in Adolescent and Young Adult Nonhuman Primates. Eneuro.

[50] Arain M., Haque M., Johal L., Mathur P., Nel W., Rais A., Sandhu R., Sharma S. (2013). Maturation of the adolescent brain. Neuropsychiatr. Dis. Treat..

[51] Seong K.J., Lee H.G., Kook M.S., Ko H.M., Jung J.Y., Kim W.J. (2016). Epigallocatechin 3 gallate rescues LpS impaired adult hippocampal neurogenesis through suppressing the TLR4 NF-κB signaling pathway in mice. Korean J. Physiol. Pharmacol..

[52] Bai Q., Lyu Z., Pan Z., Lou J., Dong T. (2017). Epigallocatechin-3-gallate promotes angiogenesis via up-regulation of Nfr2 signaling pathway in a mouse model of ischemic stroke. Behav. Brain Res..

[53] Hossain M.M., Banik N.L., Ray S.K. (2012). Survivin knockdown increased anti-cancer effects of (-)-epigallocatechin-3-gallate in human malignant neuroblastoma SK-N-BE2 and SH-SY5Y cells. Exp. Cell. Res..

[54] Vallés S., Pitarch J., Renau-Piqueras J., Guerri C. (1997). Ethanol exposure affects glial fibrillary acidic protein gene expression and transcription during rat brain development. J. Neurochem..

[55] Renno W.M., Al-Khaledi G., Mousa A., Karam S.M., Abul H., Asfar S. (2014). (-)-Epigallocatechin-3-gallate (EGCG) modulates neurological function when intravenously infused in acute and, chronically injured spinal cord of adult rats. Neuropharmacology.

[56] Koh S.H., Kim S.H., Kwon H., Park Y., Kim K.S., Song C.W., Kim J., Kim M., Yu H., Henkel J.S. (2003). Epigallocatechin gallate protects nerve growth factor differentiated PC12 cells from oxidative-radical-stress-induced apoptosis through its effect on phosphoinositide 3-kinase/Akt and glycogen synthase kinase-3. Mol. Brain Res..

[57] Biasibetti R., Tramontina A.C., Costa A.P., Dutra M.F., Quincozes-Santos A., Nardin P., Bernardi C.L., Wartchow K.M., Lunardi P.S., Gonçalves C. (2013). Green tea (-)epigallocatechin-3-gallate reverses oxidative stress and reduces acetylcholinesterase activity in a streptozotocin-induced model of dementia. Behav. Brain Res..

[58] Popova N.K., Morozova M.V., Naumenko V.S. (2011). Ameliorative effect of BDNF on prenatal ethanol and stress exposure-induced behavioral disorders. Neurosci. Lett..

[59] Liu M., Chen F., Sha L., Wang S., Tao L., Yao L., He M., Yao Z., Liu H., Zhu Z. (2014). (-)-Epigallocatechin-3-gallate ameliorates learning and memory deficits by adjusting the balance of TrkA/p75NTR signaling in APP/PS1 transgenic mice. Mol. Neurobiol..

[60] Gundimeda U., McNeill T.H., Schiffman J.E., Hinton D.R., Gopalakrishna R. (2010). Green tea polyphenols potentiate the action of nerve growth factor to induce neuritogenesis: Possible role of reactive oxygen species. J. Neurosci. Res..

[61] Feng M.J., Yan S.E., Yan Q.S. (2005). Effects of prenatal alcohol exposure on brain-derived neurotrophic factor and its receptor tyrosine kinase B in offspring. Brain Res..

[62] Haun H.L., Griffin W.C., Lopez M.F., Solomon M.G., Mulholland P.J., Woodward J.J., McGinty J.F., Ron D., Becker H.C. (2018). Increasing Brain-Derived Neurotrophic Factor (BDNF) in medial prefrontal cortex selectively reduces excessive drinking in ethanol dependent mice. Neuropharmacology.

[63] Fernández V.A., Toledano L.A., Lozano N.P., Tapia E.N., Roig M.D.G., Fornell R.D.L.T., García A.O. (2020). Bioavailability of epigallocatechin gallate administered with different nutritional strategies in healthy volunteers. Antioxidants.

